# Inequities in Access to High-Quality Home Health Agencies Among Racial and Ethnic Minorities With and Without Dementia

**DOI:** 10.1093/geroni/igab046.1276

**Published:** 2021-12-17

**Authors:** Chenjuan Ma, Bei Wu, Abraham Brody

**Affiliations:** 1 NYU, NYU Rory Meyers College of Nursing, New York, United States; 2 New York University, New York, New York, United States; 3 NYU Hartford Institute for Geriatric Nursing, New York, New York, United States

## Abstract

There are rising concerns of inequities in access to high-quality home health agencies (HHA). Using multiple national data sources that included 574,682 individuals from 8,634 HHA, we examined access to high-quality HHA care among racial and ethnic minorities with and without dementia. Approximately 9.9% of the individuals were Black, 6.2% Hispanic, and 3.3% other race/ethnicity. Over one-third (36.3%) had been diagnosed with dementia. Black and Hispanic individuals were 5.5 percentage points (95% CI, 5.2% - 5.9%) and 7.4 percentage points (95% CI, 7.0% - 7.8%) respectively more likely to receive care from agencies defined as having low-quality compared to White counterparts. Persons living with dementia were 1.3% less likely to receive care from high-quality agencies. Having dementia increased the inequity in accessing high-quality HHA between Black and White individuals. Racial and ethnic minorities, particularly those with dementia were at a disadvantaged position to receive care from high-quality HHA.

